# Predictors and Correlates of Depression in Retired Elite Level Rugby League Players

**DOI:** 10.3389/fneur.2021.655746

**Published:** 2021-04-01

**Authors:** Grant L. Iverson, Ryan Van Patten, Douglas P. Terry, Christopher R. Levi, Andrew J. Gardner

**Affiliations:** ^1^Department of Physical Medicine and Rehabilitation, Harvard Medical School, Boston, MA, United States; ^2^Spaulding Rehabilitation Hospital, Charlestown, MA, United States; ^3^Spaulding Research Institute, Charlestown, MA, United States; ^4^MassGeneral Hospital for Children Sports Concussion Program, Boston, MA, United States; ^5^Home Base, A Red Sox Foundation and Massachusetts General Hospital Program, Charlestown, MA, United States; ^6^Sydney Partnership for Health, Education, Research and Enterprise (SPHERE), Sydney, NSW, Australia; ^7^School of Medical Sciences, University of New South Wales, Randwick, NSW, Australia; ^8^Priority Research Centre for Stroke and Brain Injury, School of Medicine and Public Health, University of Newcastle, Callaghan, NSW, Australia; ^9^Hunter New England Local Health District Sports Concussion Program, Waratah, NSW, Australia

**Keywords:** retired athletes, sports, brain concussion, brain injury, depression, mental health

## Abstract

**Background:** There is considerable interest in determining whether later-in-life depression is associated with lifetime history of concussions or the duration of a career in professional contact and collision sports. Rugby league is a high-intensity collision sport involving a large number of tackles per game and a high rate of concussions. We examined predictors and correlates of depression in retired elite level rugby league players in Australia.

**Methods:** Retired elite level rugby league players (*N* = 141, age: M = 52.6, SD = 13.8; Range = 30–89 years) completed the Depression, Anxiety, and Stress Scale (DASS), Brief Pain Inventory, Connor-Davidson Resilience Scale (CD-RISC), and Epworth Sleepiness Scale; they also reported on lifetime history of concussions. The DASS depression score was regressed on age, total number of self-reported concussions, years played professionally, CD-RISC score, BPI pain interference score, and ESS score.

**Results:** The retired players reported a median of 15 total lifetime concussions [interquartile range (IQR) = 6–30], and a median of 8 years playing professional sports (IQR = 3.5–11). The proportion of the sample endorsing at least mild current depression was 29%. The DASS depression score was positively correlated with the DASS anxiety (*r* = 0.54) and DASS stress scores (*r* = 0.58). The CD-RISC score was negatively correlated with the depression score (*r* = −0.53). Depression scores were not significantly correlated with pain severity (*r* = 0.14), and were weakly correlated with life interference due to pain (*r* = 0.20) and years playing professional sports (*r* = −0.17). Depression scores were not significantly correlated with lifetime history of concussions (*r* = 0.14). A multiple regression model, with age, total number of self-reported concussions, years played professionally, the CD-RISC, Brief Pain Inventory-pain interference score, and Epworth Sleepiness Scale score as predictors was significant, with 35% of the variance in DASS depression accounted for. The two significant independent predictors of depression were lower resilience and greater life interference due to pain.

**Conclusions:** This is the first large study of depression in retired rugby league players. Depression in these retired players was not meaningfully associated with lifetime history of concussions or number of years playing elite level collision sport. Depression was associated with current anxiety, stress, resilience, and life interference due to chronic pain.

## Introduction

Some retired professional athletes suffer from depression (1–12). There is considerable interest in whether repetitive neurotrauma, either in the form of concussions, or so-called *subconcussive impacts*, is associated with later in life mental health problems in former amateur and professional contact, collision, and combat sport athletes (13–16). Researchers have reported an association between a greater number of self-reported concussions and greater symptoms of later-in-life depression (17–22). The duration of a professional career is sometimes used as a proxy for exposure to low-level repetitive mild neurotrauma (23). The literature on the association between career duration and later in life depression is mixed, with some studies reporting a positive association (24) and some not (8, 25). In recent years, researchers have asserted that depression and suicidality are clinical features of chronic traumatic encephalopathy (CTE) (26–34), although the evidence for this assertion is limited (35–39), and depression and suicidality were not considered to be clinical features of CTE between 1929 and 2009 (40, 41). Nonetheless, this assertion amplifies the importance of studying depression in retired professional contact and collision sport athletes.

### Depression in the General Population

The lifetime prevalence of depression in men born in the United States is ~20–28% (42). In the general population, it is understood that depression arises from the *cumulative* effects (43–45) of (i) genetics (46–49), (ii) adverse events in childhood (50–53) such as abuse and neglect, and (iii) current life stressors (54–57) such as relationship problems (58–60), employment instability (61–63), and financial problems (61, 62, 64, 65). Retired professional athletes can be affected by any of those factors.

### Chronic Pain and Depression

Importantly, former professional athletes also suffer from chronic pain (2, 3, 15, 66), and chronic pain is a well-established risk factor for and correlate of depression (67). Chronic pain is bidirectionally associated with depression (68–72) in that those with depression are more likely to have chronic pain and those with chronic pain are more likely to develop depression—and the two conditions might magnify each other. People with chronic pain are also at increased risk for suicidal ideation (73) and suicide (74). There is an association between chronic pain and depression in former professional soccer players (8) and NFL players (66). In former NFL players, there is an association between pain catastrophizing and depression (3). Moreover, in retired NFL players, the association between chronic pain and depression appears to be partially mediated by worsening physical functioning and other functional limitations (75). In a survey that sampled former NFL players with chronic pain, depression, or both, many reported difficulties related to their retirement transition away from football, such as financial trouble, sleep difficulties, weight gain, relationship problems, and more (2). These findings are summarized in Table 1.

**Table 1:** Transition and retirement problems in NFL players with chronic pain and depression.

**Transition and retirement problems in retired NFL players**	**Chronic** ** pain** ** (*n* = 769;** ** 47.6%)**	**Moderate or** ** severe depression** ** (*n* = 237;** ** 14.7%)**	**Chronic** ** pain and** ** depression** ** (*n* = 173;** ** 10.7%)**
Difficulty finding employment	24%	42%	45%
If employed, difficulty finding satisfaction in work	32%	52%	55%
Limited or no health insurance coverage	36%	45%	49%
Financial difficulties	34%	61%	64%
Relationship or marital problems	29%	48%	51%
Lack of social support or friendships	18%	35%	36%
Loss of a sense of being part of a community or a “part of the team”	32%	54%	57%
Trouble with transition to life after professional football	38%	59%	61%
Difficulty with pain	100%	73%	100%
Difficulty with aging	47%	58%	70%
The use of prescribed medication, alcohol, or other drugs	39%	56%	65%
Trouble sleeping	44%	74%	76%
Loss of fitness and lack of exercise	43%	69%	74%
Weight gain	41%	60%	65%

*There were 1,617 retired NFL players who completed this survey (mean age = 53.4 years, SD = 14.5) (2). For chronic pain, they endorsed the item “difficulty with pain” as “quite common” or “very common.” For moderate or severe depression, they scored 10 or greater on the PHQ-9. These data were extracted from Tables 1, 3, 4 in (2)*.

### Resilience and Depression

There is considerable interest in the role resilience might play regarding both depression and chronic pain (76–80). Resilience underlies a person's ability to cope with, adapt to, or overcome adversity. Resilience is manifested through adaptive behavior (81), a positive view of one's self, the world, and the future (82), and positive emotions, even in stressful situations (83). Greater resilience is associated with fewer symptoms of depression in older adults in general (84), less severe self-reported depression in older adults who are experiencing major depressive disorder (85), and a greater response to medication treatment for depression (86).

### Purpose and Hypotheses

For the past 8 years, a study of the brain health of retired elite level rugby league players from Australia has been underway (87–90). This study was approved by the Institutional Human Ethics Committee. Rugby league is a high-intensity collision sport involving a large number of tackles per game (91, 92) and a fairly high rate of concussions (93). The purpose of the current study is to examine predictors and correlates of depression in retired elite level rugby league players. We hypothesized that (i) a small subgroup of retired players would endorse symptoms consistent with depression. In terms of simple bivariate relationships, we hypothesized that (ii) a greater number of past concussions would be associated with more symptoms of current depression, (iii) more years of exposure to professional sport would be associated with more symptoms of depression, (iv) greater resilience would be associated with fewer symptoms of depression, and (v) greater chronic pain would be associated with more symptoms of depression. However, these variables are likely interrelated in retired elite level rugby league players, and we were ultimately interested in the strongest overall predictor(s) of depressive symptoms among the larger set of correlates. Consequently, in a multivariate model predicting depression from the following variables: age, lifetime history of concussions, years playing professional rugby league, resilience, chronic pain severity, and subjective sleepiness, we hypothesized that only resilience and chronic pain severity would be significant independent predictors.

## Materials and Methods

### Participants

Participants were retired elite level rugby league players. They included players that competed at the first grade level in the New South Wales Rugby League (NSWRL, 1908–1994), Australian Rugby League (1995–1997), Super League (1997), and/or National Rugby League (1998–present). These men were recruited in two ways. First, they were recruited through communication *via* club “old boys” (alumni) networks, who distributed study information to their members. Second, they were recruited through direct and indirect referrals from the National Rugby League and the Men of League Foundation, who also distributed information pertaining to the study to their members. Potential participants were excluded if they reported a history of neurosurgery or of a brain tumor requiring radiation treatment. We analyzed the data from the first 145 retired players. For this study, we excluded four of these men because they did not complete the self-report measure of depression (three of whom had dementia, which precluded their full participation in the study).

### Procedures

This study, called *The Retired Professional Rugby League Players Brain Health Research Program*, has been running since 2012, and has been modified over the years. In its present form, the study incorporates a health survey, an in-person clinical evaluation, multi-modal experimental neuroimaging, blood biomarkers and genetic testing, and brain and spinal cord donation. Participants may opt in or out of any aspect of the research protocol. All participants completed a clinical interview, which was immediately followed by neurocognitive testing with a clinical neuropsychologist (i.e., author AG). The clinical interview collected data pertaining to demographic information and medical and concussion history, together with patient reported outcome measures. The interview and neurocognitive testing took ~2 h and 30 min in total. All variables used in this study were collected during the clinical interview and on questionnaires.

### Measures

#### Exposure Variables

Concussion history was based on self-report. During an interview, a definition of concussion was provided to the participants (from the Consensus statement on concussion in sport, 2012 definition) (94). Participants were allowed to ask questions in order to clarify the definition. They reported the number of sport-related and non-sport-related concussions they sustained throughout their lives; these two values were summed in order to determine the total number of lifetime concussions for each participant. This variable was used for all lifetime concussion analyses in this manuscript. Number of years played at the elite level and number of games played at the elite level were also based on self-report.

#### Questionnaires

The Depression, Anxiety, Stress Scale 21-item (DASS-21) (95) is a shortened version of the original 42-item version (96). Each item is a statement about a negative emotional symptom and is rated by the participant on a 0–3 scale based on the extent to which they experienced that symptom over the past week. The scores for each of the three scales (i.e., depression, anxiety, and stress) are derived by summing the scores for the seven items that comprise that scale and multiplying the sum by 2. Higher scores reflect more symptoms. The total score for each scale is converted to a classification (i.e., normal, mild, moderate, severe/extremely severe) using the DASS Manual (97). Internal consistency (Cronbach's alpha) is high for each scale (depression = 0.94, anxiety = 0.87, Stress = 0.91) (95).

The Connor-Davidson Resilience Scale (CD-RISC) (98) is a 25-item measure that evaluates resilience without specifying a particular time interval. Each item is rated on a 0–4 scale (i.e., “not at all true” to “true nearly all of the time”), with a total score range from 0 to 100. Higher scores reflect greater resilience. Factor analysis of this scale yielded five factors, which were broadly interpreted as (1) personal competence/tenacity, (2) trust in one's instincts/tolerance of negative affect, (3) positive acceptance of change, (4) control, and (5) spiritual influences. It has high internal consistency (Cronbach's alpha = 0.89) in the general population (98).

The Epworth Sleepiness Scale (ESS) (99) is an 8-item self-report scale that assesses how likely the participant is to fall asleep during a variety of different situations (e.g., “sitting and reading,” “watching TV”). There is no specified time interval for the participant to consider. Each item is rated on a 0–3 scale (i.e., would never doze, slight chance of dozing, moderate change of dozing, high chance of dozing), with a total score range of 0–24. The questionnaire has a high level of internal consistency (Cronbach's alpha = 0.88) (100). Test-retest reliability (i.e., intra-class correlation coefficient) was 0.88 in a large sample of high school students (101).

The Brief Pain Inventory (BPI), Short Form (102, 103) is a self-report scale that measures the severity of pain and how it has interfered with activities over the past week. One subscale measures Pain Severity, which is the average of four items that assess pain at its worst, least, on average, and currently; each item is rated 0–10, from “no pain” to “pain as bad as you can imagine.” The second subscale measures Pain Interference, which is the average of seven items that assess the degree to which the pain interferes with general activity, mood, walking ability, sleep, etc.; each item is rated 0–10, from “does not interfere” to “completely interferes.” Higher scores indicate greater severity/interference. Internal consistency and test-retest reliability are consistently high across studies (104).

The Alcohol Use Disorders Identification Test (AUDIT): Self-Report Version (105, 106) is a 10-item instrument developed by the World Health Organization to identify harmful alcohol consumption by assessing frequency/amount of alcohol use (Questions 1–3), alcohol dependence (Questions 4–6), and alcohol-related problems (Questions 7–10). Various time intervals are assessed (e.g., weekly use of alcohol, consumption in the past year). Each item is rated on a 0–4 scale, with score ranges from 0 to 40 (higher = more hazardous alcohol use). A systematic review of its psychometric properties indicated a test-retest reliability over 0.8 on most studies, as well as a Cronbach's alpha of 0.81–0.93 (107). Risk level was classified based on the total score (0–7 = low risk; 8–15 = risky/hazardous; 16–19 = high-risk/harmful; ≥20 = high-risk) based on the manual (106).

### Statistical Analyses

Descriptive statistics were computed for all variables used in the analyses. Most variables were non-normally distributed, so non-parametric statistics were used where appropriate. Spearman correlations were conducted between DASS depression, anxiety, and stress scores, as well as between these three variables and (i) the CD-RISC score, (ii) the BPI pain severity and pain interference scores, (iii) the AUDIT score, and (iv) the ESS score. Spearman correlations were also conducted between the DASS depression score and (i) total number of self-reported concussions and (ii) contact sport exposure (years playing rugby professionally and number of professional games played).

A simultaneous multiple regression was modeled, with the DASS depression score as the outcome and the following variables as predictors: age, total number of self-reported concussions, years played professionally, CD-RISC score, BPI pain interference score, and ESS score. Plots of standardized predicted values against standardized residuals were visually inspected to evaluate normality and homoscedasticity. Standardized residuals (with values ±1.96 reflecting unusual cases) and Cook's distance (with >1 defining problematic influential cases) were used to examine outliers. Multicollinearity was defined as correlations among predictors >0.8, variance inflation factor (VIF) values >10, or tolerance values <0.1. Model cross-validation was assessed by examining adjusted *R*^2^ in addition to standard *R*^2^. Data were analyzed with SPSS version 27.0.

For the hypothesis-based correlational analyses, we used an online calculator to compute the False Discovery Rate. This method controls for Type I error, with alpha set at *p* < 0.05 (two-tailed tests), which decreases the odds of finding a false positive result while maintaining high statistical power compared to other methods used to correct for multiple comparisons [e.g., Bonferroni correction; (108)].

## Results

### Demographic Characteristics and Exposure History

The mean age of the sample was 52.6 years (SD = 13.8, Range = 30–89) and the mean years of education was 11.9 (SD = 2.6, Range = 7.0–18.0). Approximately 71.6% of the retired players were married, 17.0% were divorced, 7.8% reported their marital status as single, 2.1% were cohabitating, and 1.4% were separated. The retired players reported their employment status as follows: employed full-time = 77.3%, employed part-time = 2.8%, retired = 18.4%, or unemployed = 1.4%. The median number of past concussions reported was 15.0, with 50% of the sample reporting between 6.0 and 30.0 prior concussions (Table 2). The median number of years competing at the elite level was 8.0 (IQR = 3.5–11.0).

**Table 2:** Self-reported exposure history.

	***n***	**Mean**	**SD**	**Md**	**IQR**	**Range**
Self-reported concussion history	141	28.2	36.4	15.0	6.0–30.0	0.0–200.0
Number of concussions with LOC	141	4.4	6.3	3.0	1.0–5.5	0.0–50.0
Years played professionally	141	8.0	4.7	8.0	3.5–11.0	1.0–20.0
Games played professionally	138	121.7	100.7	94.5	21.8–210.8	1.0–357.0

*LOC, loss of consciousness; n, sample size; SD, standard deviation; Md, median; IQR, interquartile range*.

### Health History and Self-Report Measures

Descriptive statistics for the self-report measures are provided in Table 3. The mean scores for the sample on the depression, anxiety, and stress measures (DASS) were in the “normal classification range.” A lifetime history of depression was reported by 28.4% of the sample (Table 4). A lifetime history of drug use was reported by 28.4%. Regarding current psychological distress on the DASS, 29.1% of the retired players reported at least mild depression, 19.2% reported at least mild anxiety, and 27.0% reported at least mild stress. The breakdown of participants by DASS Depression classification ranges was as follows: Broadly Normal = 70.9%, Mild = 14.9%, Moderate = 9.9%, and Severe/Extremely Severe = 4.3%.

**Table 3:** Self-report measures.

	***n***	**Mean**	**SD**	**Md**	**IQR**	**Range**
DASS depression	141	6.5	7.9	4.0	0.0–10.0	0.0–42.0
DASS anxiety	141	4.1	5.4	2.0	0.0–6.0	0.0–32.0
DASS stress	141	10.0	9.1	8.0	2.0–15.0	0.0–42.0
Alcohol use (AUDIT)	141	7.5	5.0	6.0	4.0–11.0	0.0–22.0
Resilience (CD-RISC)	133	74.6	14.7	77.0	67.0–85.0	38.0–100.0
Epworth sleepiness scale	141	7.0	5.0	6.0	3.0–10.0	0.0–24.0
Pain severity (BPI)	133	2.1	2.1	1.8	0.0–3.8	0.0–7.3
Pain interference (BPI)	133	1.8	2.3	0.6	0.0–3.4	0.0–8.4

*n, sample size; SD, standard deviation; Md, median; IQR, interquartile range; AUDIT, Alcohol Use Disorders Identification Test; BPI, Brief Pain Inventory; CD-RISC, Connor-Davidson Resilience Scale; DASS, Depression, Anxiety, Stress Scales; DASS, Depression Normal range = 0–9; Anxiety Normal range = 0–7; Stress Normal range = 0–14*.

**Table 4:** Past and present health problems.

**Current or recent…**		**Lifetime history of…**	
Depression (DASS)		Depression	40 (28.4%)
Normal (0–9)	100 (70.9%)	Psychiatric history	27 (19.1%)
Mild (10–13)	21 (14.9%)	Lifetime illicit drug use	40 (28.4%)
Moderate (14–20)	14 (9.9%)	Headache	35 (24.8%)
Severe/extremely severe (21+)	6 (4.3%)	Migraine	15 (10.6%)
Anxiety (DASS)		Sleep apnea	8 (5.7%)
Normal (0–7)	114 (80.9%)	Epilepsy	3 (2.1%)
Mild (8–9)	11 (7.8%)	Stroke	5 (3.5%)
Moderate (10–14)	9 (6.4%)	Family history of dementia	45 (31.9%)
Severe/extremely severe (15+)	7 (5.0%)	Hypertension	19 (13.5%)
Stress (DASS)		High cholesterol	12 (8.5%)
Normal (0–14)	103 (73.0%)	Myocardial infarction	3 (2.1%)
Mild (15–18)	16 (11.3%)	Other heart condition	6 (4.3%)
Moderate (19–25)	13 (9.2%)	Peripheral vascular disease	4 (2.8%)
Severe/extremely severe (26+)	9 (6.4%)	COPD	3 (2.1%)
Alcohol use (AUDIT classification)		Diabetes	8 (5.7%)
Low-risk	78 (55.3%)	Thyroid disease	2 (1.4%)
Risky or hazardous level	53 (37.6%)	Liver disease	4 (2.8%)
High-risk or harmful level	7 (5.0%)	Renal insufficiency	2 (1.4%)
High-risk (likely dependence)	3 (2.1%)	Peptic ulcer	7 (5.0%)
Recent cannabis use (past 6 months)	7 (5.0%)	Cancer	13 (9.2%)
Recent illicit drug use (past 6 months)	18 (12.8%)	Arthritis	71 (50.4%)

*AUDIT, Alcohol Use Disorders Identification Test; COPD, Chronic obstructive pulmonary disease; DASS, Depression, Anxiety, Stress Scales. Psychiatric history refers other mental health problems not including depression. Of the 27 men who reported other mental health problems, 24 also endorsed a history of depression*.

### Bivariate Predictors of, and Correlates With, Depression

The DASS depression, anxiety, and stress subscales were positively correlated with one another (*r* = 0.54–0.58). Correlations between DASS depression, anxiety, and stress scores and other variables of interest are presented in Table 5.

**Table 5:** Bivariate Spearman correlations.

	**Depression**	**Anxiety**	**Stress**
Resilience (CD-RISC)	−0.53^a^	−0.33^a^	−0.31^a^
Pain severity (BPI)	0.14	0.16	0.21^c^
Pain interference (BPI)	0.20^c^	0.20^c^	0.24^b^
Alcohol use (AUDIT)	−0.10	−0.08	−0.06
Epworth sleepiness scale	−0.05	0.00	−0.13
Number of self-reported concussions	0.14	0.09	0.23^b^
Years played professionally	−0.17^c^	−0.14	−0.11
Games played professionally	−0.20^c^	−0.18^c^	−0.12

*AUDIT, Alcohol Use Disorders Identification Test; BPI, Brief Pain Inventory; CD-RISC, Connor-Davidson Resilience Scale; DASS, Depression, Anxiety, Stress Scales*.

a *p < 0.001*,

b p < 0.01, and

c *p < 0.05*.

*The correlation between years of exposure to professional sports and depression was not significant after applying the FDR adjustment to the p-value (FDR p = 0.07). The correlation between life interference due to pain and depression was not significant after applying the FDR adjustment to the p-value (FDR p = 0.06)*.

Hypotheses (ii)–(v) relate to the associations between symptoms of depression and concussion history, years of exposure to professional sports, resilience, and chronic pain (both severity and life interference). There was no significant correlation between lifetime history of concussions and depression. There was a small significant *negative* correlation (*r* = −0.17, *p* = 0.04) between years of exposure to professional sports and depression (although this was not significant after applying the FDR *p*-value adjustment for the five hypothesis-based correlations: FDR *p* = 0.07). The resilience score (i.e., CD-RISC) was negatively correlated with depression (*r* = −0.53; *p* < 0.001), with a large effect size, indicating that higher levels of resilience are related to lower depression. Pain severity was not significantly correlated with depression. There was a small, significant (*r* = *0.2*0; *p* = 0.02) correlation between life interference due to pain and depression, but this association was not significant after applying the FDR adjustment to the *p*-value (FDR *p* = 0.06).

### Multivariable Prediction Model

When evaluating the statistical assumptions for the regression, no Cook's distances exceeded 1. Only five cases (3.5% of the sample) had standardized residuals of ±1.96, suggesting that influential cases were not an issue in the model. Multicollinearity was also not a problem because the correlations between predictors were all well below 0.8, VIF values were all <1.1, and tolerance values were all >0.95. Assumptions of normality and homoscedasticity may not have been met for all variables, based on plots of standardized predicted values against standardized residuals. However, results from bootstrapped parameters (1,000 samples) did not differ from the initial results, suggesting no undue influence on model parameters.

Results of the regression are presented in Table 6. The model was significant (*p* < 0.001), with 35% of the variance in DASS depression accounted for by the set of predictors. After adjusting for model cross-validation, *R*^2^ was reduced to 0.32. Consistent with hypothesis (vi), the only significant predictors of DASS depression were resilience (the CD-RISC), β = −0.51, *t* = −6.97, *p* < 0.001 and BPI pain interference, β = 0.21, *t* = 2.83, *p* < 0.01. Age, number of self-reported concussions, years played at the elite level, and sleepiness were not significant.

**Table 6:** Simultaneous regression model with DASS depression as the outcome.

	**B**	**SE B**	**β**	***t* (*p*)**	**Bootstrapped** ** 95% CI** ** for B**
Constant	24.81	4.78	-	–	–
Age	−0.02	0.04	−0.04	−0.50 (0.62)	−0.10, 0.07
Number of self-reported concussions	0.02	0.02	0.07	1.00 (0.35)	−0.02, 0.04
Years played professionally	0.11	0.11	0.08	1.03 (0.30)	−0.14, 0.34
Resilience (CD-RISC)	−0.28	0.04	−0.51	−6.97 (<0.001)	−0.40, −0.17
Epworth sleepiness scale	−0.09	0.12	−0.06	−0.77 (0.45)	−0.30, 0.12
Brief pain inventory interference	0.72	0.25	0.21	2.83 (0.01)	0.14, 1.36

*R^2^ = 0.35, F_(6, 126)_ = 11.26, p < 0.001; Adjusted R^2^ = 0.32. B, unstandardized regression coefficient; β, standardized regression coefficient; CD-RISC, Connor-Davidson Resilience Scale; CI, Confidence Interval; DASS, Depression, Anxiety, Stress Scales; SE, Standard Error. Decisions regarding statistical significance of the individual predictors did not differ after the implementation of the FDR correction; uncorrected p-values are presented in the table*.

## Discussion

We examined predictors and correlates of depression in retired elite level rugby league players. A lifetime history of depression was reported by 28.4% of the sample. Half of the retired players reported having arthritis (50.4%). Some degree of alcohol misuse was reported by 44.7% of the sample, and 12.8% reported recent drug use. Current moderate to severe depression (14.8%), anxiety (11.4%), and stress (15.6%) was reported by a small percentage of the sample. Greater resilience was associated with lower levels of depression, anxiety, and stress. There was a small positive correlation between life interference due to pain and depression. There was no association between lifetime history of concussions and current depression. There was a very weak *negative* correlation between exposure to professional sports (years played and games played) and current symptoms of depression, meaning that greater exposure was associated with fewer symptoms of depression. Finally, a multivariate model regressing depression on age, lifetime history of concussions, years playing elite rugby league, resilience, life interference due to chronic pain, and sleepiness accounted for 35% of the variance in depression. The only statistically significant independent predictors of depression were resilience and life interference due to pain.

### Correlates of Depression in Former Elite and Professional Athletes

Some former professional contact and collision sport athletes experience depression (1–4, 7–12, 109). There are many reasons why former professional athletes might experience depression. The transition to retirement is often difficult and it can be associated with a tremendous sense of loss (110), a void in meaning and purpose (111, 112), and even financial hardship (113). Those former athletes who retire due to injury tend to have more difficulty with psychological adjustment to retirement than those who are more in control of their retirement decision (8). Most elite athletes have a strong sense of athletic identity (8, 114), which refers to the degree to which they identify very strongly with their athlete role as a core part of their fundamental sense of self, and look to others for acknowledgment and validation of that identity. Greater athletic identity has been associated with greater symptoms of depression in some retired professional athletes (8). In a study of former professional soccer players, screening positively for depression was associated with being younger, more recently retired, having higher athletic identity, and citing injury as the main reason for retirement (8). That said, in large-scale cohort study comparing former professional soccer players (*n* = 7,676) to matched general population control subjects (*n* = 23,028), hospital admissions for common mental health disorders were lower for the former players than the population controls (115).

Chronic pain (2) and osteoarthritis (116) are associated with depression and psychological distress in elite and professional athletes. In the present study, half (50.4%) reported arthritis and we found a weak association that approached significance between life interference due to chronic pain and current symptoms of depression. In the general population, there is a strong association between chronic pain and depression (67–72). In studies of retired NFL players (2, 3, 66, 75) and former athletes in other contact sports (8), chronic pain is associated with symptoms of depression. In one study of former NFL players (3), the correlation between symptoms of depression, measured using the CES-D, and pain intensity (*r* = 0.43) and life interference due to pain (*r* = 0.58) were considerably higher than correlations in the present study between symptoms of depression measured on the DASS and pain severity (*r* = 0.14) and life interference due to pain (*r* = 0.29).

In the present study, we found a strong negative association between resilience and depression. Although prior studies with retired elite and professional athletes have examined the prevalence and correlates of depression (15) and common mental disorders (9), the association between resilience and depression has not, to our knowledge, been explored. Nonetheless, researchers have recommended developing interventions for retired athletes that improve psychological resilience (9). In the general population, greater resilience is associated with fewer symptoms of depression in older adults (84), less severe self-reported depression in older adults who are experiencing major depressive disorder (85), and a greater response to medication treatment for depression (86).

### Association Between Depression and Exposure to Neurotrauma

In the present study, a greater lifetime history of concussions and a greater history of exposure to elite-level collision sports were not associated with current symptoms of depression. This study is inconsistent with survey studies of former elite and professional athletes that have shown an association between a greater number of prior concussions and current symptoms of depression (17–22). Those studies were conducted prior to the greatly increased awareness of, and sensitivity to, concussions. Additionally, subjects in those studies reported far fewer past concussions than the subjects in the present study, and the effects of this reporting difference are unknown. Our results are also inconsistent with a small clinical study of retired NFL players that showed a significant correlation of *r* = 0.43 between lifetime number of concussions and Beck Depression Inventory-Second Edition scores (117). The results of this study also differ from other clinical studies of retired NFL players that have shown an association between a greater number of years played professionally and current symptoms of depression (24). These results align, however, with other studies of retired elite or professional athletes that have not found an association between concussion history (118) or greater number of years of exposure (8, 25) and current symptoms of depression or psychological distress.

### Limitations

This study has several limitations. First, the study design was cross sectional and, therefore, no casual inference is permitted. Second, the sample consisted of male retired professional rugby league players, a narrow demographic group, which limits generalizability to other populations (e.g., women, amateur athletes, athletes from other sports). Third, it is possible that sampling bias may have led to the recruitment of healthier and higher functioning participants because they are more likely to be connected with alumni organizations and were informed about this research. Fourth, both lifetime concussion history and contact sport exposure were assessed *via* self-report. Consequently, these data may be subject to biases and memory failures associated with retrospective recall. Fifth, the various self-report measures used in this study assessed symptoms over somewhat different time intervals. Therefore, it was not possible to evaluate long-standing (or lifetime) health issues for those variables that only evaluated a more recent time interval. For example, the DASS and BPI measure symptoms of depression, anxiety, stress, and pain over the past week, and the CD-RISC and ESS evaluate resilience and sleepiness in general, without specifying a particular time period. There may be limited associations among the variables, given that psychological and other health factors can fluctuate over time. Sixth, we did not use structured psychiatric interviews to assess the full criteria for psychiatric disorders. Finally, the goal of this study was to examine the association between indices of past neurotrauma (i.e., history of concussions and duration of exposure to elite level sports) and current depressive symptoms. We included a small number of other variables that have previously been associated with depression symptoms. Our goal was not to try to comprehensively explain the variance in depressive symptoms, and we did not include other variables in the model that may be related to depressive symptoms such as alcohol/drug use, or disability status. A future study with a larger sample size could include more demographic and clinical variables in the model.

### Conclusions

Researchers have reported that correlates of depression and psychological distress in retired elite and professional athletes include retirement due to injury (8), greater athletic identity (8), a higher level of career dissatisfaction (11, 119), current adverse life events (11, 12, 119), chronic pain (2, 3, 66, 75), osteoarthritis (116), and lifetime history of prior concussions (17–22). In the present study, neither lifetime history of concussions nor contact sport exposure were meaningfully related to depression in retired elite level rugby league players. Resilience was found to be a particularly strong correlate of depression. Future researchers should examine longitudinal predictors of depression in order to better delineate the developmental trajectory of depression during and after rugby league participation.

## Data Availability Statement

The original contributions presented in the study are included in the article, further inquiries can be directed to the corresponding author. The statistical code, syntax, output, and analyses are available to qualified researchers upon request. Requests for this information should be directed to Andrew J. Gardner, Andrew.Gardner@newcastle.edu.au.

## Ethics Statement

The studies involving human participants were reviewed and approved by the University of Newcastle Human Ethics Committee. The patients/participants provided their written informed consent to participate in this study.

## Author Contributions

GI conceptualized the study and wrote portions of the manuscript. RV, GI, and DT conceptualized the statistical analyses. RV performed the statistical analyses and prepared the first draft of the Results section. CL assisted with securing funding. AG is the overall principal investigator, secured funding for the larger parent study, directed that study, collected all of the data, managed the database, helped conceptualize this study, and wrote portions of the manuscript. All authors critically reviewed the manuscript, read, and approved the last version of this manuscript.

## Conflict of Interest

GI serves as a scientific advisor for NanoDx™ (formerly BioDirection, Inc.), Sway Operations, LLC, and Highmark, Inc. He has a clinical and consulting practice in forensic neuropsychology, including expert testimony, involving individuals who have sustained mild TBIs (including athletes). He has received research funding from several test publishing companies, including ImPACT Applications, Inc., CNS Vital Signs, and Psychological Assessment Resources (PAR, Inc.). He has received research funding as a principal investigator from the National Football League, and salary support as a collaborator from the Harvard Integrated Program to Protect and Improve the Health of National Football League Players Association Members. DT serves as a consultant for REACT Neuro, Inc. CL serves as a consultant neurologist to the National Rugby League (NRL) providing a pro bono second opinion to current players regarding concussion assessment and management for the player's club doctor. AG serves as a scientific advisor for hitIQ, Ltd. He has a clinical practice in neuropsychology involving individuals who have sustained sport-related concussion (including current and former athletes). He has been a contracted concussion consultant to Rugby Australia since July 2016. He has received travel funding or been reimbursed by professional sporting bodies, and commercial organizations for discussing or presenting sport-related concussion research at meetings, scientific conferences, workshops, and symposiums. Previous grant funding includes the NSW Sporting Injuries Committee, the Brain Foundation (Australia), an Australian-American Fulbright Commission Postdoctoral Award, a Hunter New England Local Health District, Research, Innovation and Partnerships Health Research & Translation Centre and Clinical Research Fellowship Scheme, and the Hunter Medical Research Institute (HMRI), supported by Jennie Thomas, and the HMRI, supported by Anne Greaves. He is currently funded through an NHMRC Early Career Fellowship, and the University of Newcastle's Priority Research Centre for Stroke and Brain Injury. He has received research funding from the National Rugby League (NRL) for the Retired Players Brain Health research program. The remaining author declares that the research was conducted in the absence of any commercial or financial relationships that could be construed as a potential conflict of interest.
